# Evaluating large language models for criterion-based grading from agreement to consistency

**DOI:** 10.1038/s41539-024-00291-1

**Published:** 2024-12-30

**Authors:** Da-Wei Zhang, Melissa Boey, Yan Yu Tan, Alexis Hoh Sheng Jia

**Affiliations:** Department of Psychology, Jeffrey Cheah School of Medicine and Health Sciences, Monash University Malaysia, Bandar Sunway, 475000 Malaysia

**Keywords:** Psychology, Education

## Abstract

This study evaluates the ability of large language models (LLMs) to deliver criterion-based grading and examines the impact of prompt engineering with detailed criteria on grading. Using well-established human benchmarks and quantitative analyses, we found that even free LLMs achieve criterion-based grading with a detailed understanding of the criteria, underscoring the importance of domain-specific understanding over model complexity. These findings highlight the potential of LLMs to deliver scalable educational feedback.

The emerging abilities of large public language models (LLMs) in various domains have aroused vigorous discussion in education. Recent surveys reveal that LLMs (e.g., ChatGPT) are widely used by students at various academic levels^1^. This prevalence, however, raises concerns about LLMs’ overreliance leading to avoidance of learning^2^. Yet, given the transformative potential of LLMs in education, dismissing their use is unwise; instead, it is essential to explore accountable ways to integrate LLMs into educational practices^2,3^.

One promise of integrating LLMs into education lies in its capacity to provide feedback – a core educative process for promoting learning. Despite the importance of feedback, students often experience insufficient or ineffective feedback during learning^4,5^. Feedback is a constructive process of providing comments to bridge the gap between a learner’s current understanding and learning objectives^6^. Providing effective feedback requires expertise, quick response time, interactive capabilities, and an objective attitude^5^. LLMs possesses all these features, positioning themselves as a promising agent for providing feedback. However, integrating LLMs as a feedback agent in education needs empirical validation of its effectiveness^3^.

Recent empirical research demonstrates that LLMs can provide effective feedback from the perspective of feedback recipients. The effectiveness of feedback depends on its reception. In contrast to human raters, ChatGPT offers more intricate and encouraging feedback on students’ essays, potentially offsetting human-rater limitations^7^. Student feedback surveys indicate a favourable reception towards feedback generated by ChatGPT^8–10^. An experimental study further corroborates these findings, revealing a considerable preference among students for ChatGPT-generated feedback because of its clarity and specificity^11^. Similar results were reported in an interventional study where ChatGPT-generated feedback improved learning engagement and motivation during writing training^12^; however, its quality might vary with the complexity of writing^13^. Besides writing tasks, LLMs, such as ChatGPT and Bing chat, can also generate content-based feedback for medical multiple-choice exams, demonstrating the potential for using LLMs to provide scalable and structured feedback^14^.

A critical issue remains regarding the extent to which LLM-generated feedback is criterion-based. Identifying the discrepancy between a learner’s status and the desired learning outcomes is an essential feature of effective feedback^6^. However, LLM-generated content is generally prone to illogical reasoning and unsupported assumptions^2^, raising concerns about its reliability in providing feedback^7^. While recent studies suggest that LLMs, such as ChatGPT and Bard, can align their feedback with predefined criteria to some extent^13,15^, significant concerns persist due to methodological limitations. These studies primarily focus on broad metrics, such as overall score magnitude (e.g., higher or lower) and sentiment polarity (positive/negative) compared to human evaluators, failing to capture the nuance of how well the feedback addresses the criteria. Furthermore, reliance on expert evaluations, where experts may know which feedback was generated by LLMs, risks introducing bias. Questions also remain about the validity of human benchmarks and the learning criteria used in these comparisons. These limitations underscore the need for more detailed evaluations.

A nuanced way to evaluate how well LLMs respond to criteria is through grades. Criterion-based grading, a widely used form of summative feedback, assigns learners to performance levels based on predefined criteria rather than normative comparisons^16^. This approach fulfills the fundamental purpose of feedback by clarifying the gap between a learner’s current performance and desired outcomes^16^. From a research perspective, the quantitative nature of grades offers a distinct advantage, enabling systematic assessment of capacity of LLMs to deliver criterion-based feedback.

The current study uses well-established human benchmarks and quantitative methods to fill previous gaps by evaluating the extent to which LLM-generated feedback aligns with established criteria. We focus on the International English Language Testing System (IELTS) academic writing task for two primary reasons that address previous limitations: (1) it employs a well-established evaluation rubric, and (2) it provides official writing samples with corresponding grades based on these criteria. These features ensure reliable and criterion-based benchmarks for accurate evaluation. Additionally, IELTS is a widely recognized testing scenario where there should be sufficient corpus for LLMs. To gauge how LLMs align with criteria, we employ various measures of the Intraclass Correlation Coefficient (ICC), including interrater absolute agreement and interrater consistency, to capture different aspects of feedback quality and potential biases. Interrater absolute agreement evaluates the exact match in ratings between raters, while interrater consistency measures the extent to which raters maintain consistent differences in their evaluations^17^. Furthermore, we manipulate prompts to assess whether including evaluation criteria enhances the grading quality, thereby providing insights into prompt engineering for generating criterion-based feedback.

The interrater agreement and interrater consistency of each prompt are shown in Table 1. Only prompt 3 showed significant interrater agreement but at a moderate level. Regarding interrater consistency, all prompts showed significance, but again, it was Prompt 3 that achieved a good level.

**Table 1:** Interrater agreement and consistency under the three prompts

	Prompt 1	Prompt 2	Prompt 3
Interrater agreement	0.46 [−0.04, 0.74]	0.37 [−0.10, 0.73]	0.61 [0.02, 0.85]
Interrater consistency	0.63 [0.35, 0.80]	0.71 [0.48, 0.85]	0.77 [0.57, 0.88]

Point estimates and their 95% confidence intervals are presented.

Despite being the most effective, the discrepancy between the interrater agreement and consistency in Prompt 3 suggests potential systematic biases. The Bland-Altman plot showed that the biases were evenly distributed across different levels of essays (Fig. 1). However, the grading of Prompt 3 was significantly lower than that of IELTS examiners, t(60) = −2.81, p < 0.01, Cohen’s d = 0.71.

**Fig. 1:**
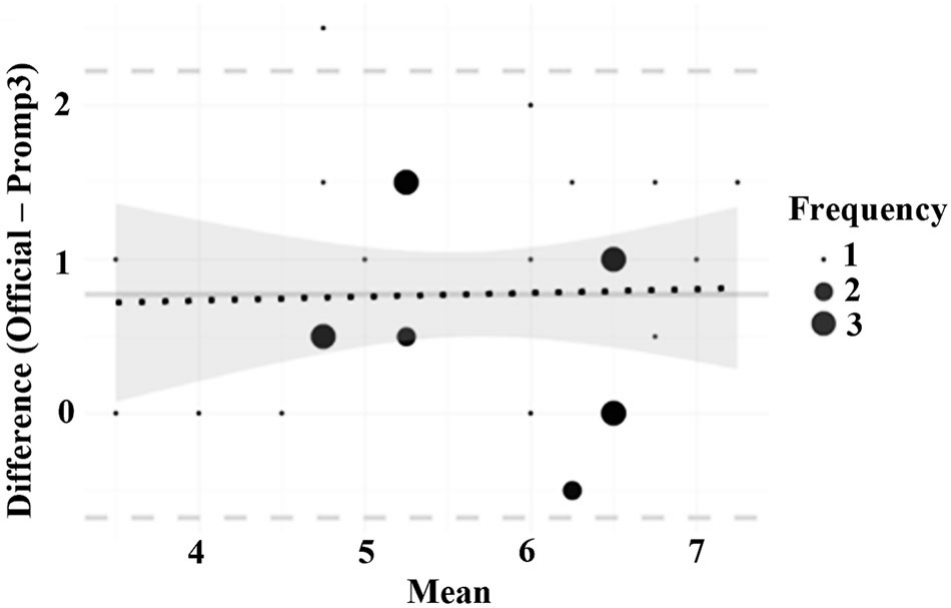
Bland-Altman plot showing agreement between IELTS official examiners and ChatGPT. The plots display the mean scores versus the differences in scores. The solid line represents the mean difference, the dashed lines represent the upper and lower limits of agreement, and the dotted line represents the trend.

In follow-up analyses, Prompt 3 exhibited good intrarater consistency (ICC = 0.89, 95% CI [0.76, 0.94]). We then reran this prompt using ChatGPT 4.0, resulting in moderate interrater agreement (ICC = 0.74, 95% CI [0.20, 0.90]) and good interrater consistency (ICC = 0.84, 95% CI [0.69, 0.92]). Qualitatively, these results were comparable to ChatGPT 3.5, achieving the same level of performance. Quantitatively, there was no significant difference from ChatGPT 3.5, as indicated by nonsignificant Fisher’s Z scores (*p* > 0.05). Applying the same prompt to Claude 3.0 Haiku yielded comparable interrater agreement of ICC = 0.68 (95% CI [0.44, 0.83]) and interrater consistency of ICC = 0.72 (95% CI [0.50, 0.86]). In contrast, the teacher’s grading demonstrated poor interrater agreement (ICC = 0.37, 95% CI [0.02, 0.63]) and poor interrater consistency (ICC = 0.44, 95% CI [0.00, 0.72]). The interrater consistency was significantly different from that generated by the best prompt condition in ChatGPT 3.5 (*p* < 0.05).

This study systematically evaluated the capability of LLMs to perform criterion-based grading and examined how prompt engineering with detailed evaluation criteria influences grading. The observed patterns of ICC measures across various conditions confirm that LLMs can provide reliable and criterion-based grading, providing theoretical and practical insights for the effective integration of LLMs into educational feedback practices.

Our findings indicate that domain-specific knowledge, rather than the complexity or general knowledge/abilities of the LLMs, limits the precision of criterion-based grading. We selected the IELTS writing task due to its highly structured nature and the extensive corpus available, which should theoretically enable LLMs to provide precise grading. Nevertheless, the most optimized prompt only achieved moderate absolute agreement and significantly lower grades compared to official marking, suggesting systematic biases and raising concerns about using the absolute grading provided by LLMs such as ChatGPT and Claude. We speculate that the suboptimal agreement may result from a lack of domain-specific knowledge rather than the models’ complexity or general knowledge/abilities. Firstly, the most accurate grading in our study was derived from the prompt that involved the most domain-specific knowledge (i.e., detailed marking criteria in this case). Secondly, using more advanced models (i.e., ChatGPT 4.0 and Claude 3.0), possessing more superior domain-general knowledge and general abilities compared to ChatGPT 3.5, did not quantitatively or qualitatively improve grading.

Meanwhile, our results confirm that LLMs can deliver reliable and criterion-based grading. Equipped with detailed knowledge of criteria, LLMs achieved good interrater consistency with benchmarks and demonstrated excellent test-retest reliability. These findings highlight the efficacy of LLMs in adhering to established grading standards and emphasize the critical role of domain-specific knowledge in enhancing grading performance. Coupled with the previously discussed suboptimal absolute grading, the reliable and criterion-based grading provided by LLMs suggests that responsible use should focus not on one-time grades but on tracking learning growth against desired criteria. This approach effectively uses criterion-based grading as meaningful educational feedback for learning^16^.

This study encourages further empirical studies on integrating LLMs into educational feedback practices. Identifying performance gaps using established criteria is essential for providing effective educational feedback^6^. While this study suggests that LLMs equipped with domain-specific knowledge can identify the gap, future research could extend this work by examining the ability of LLMs to generate more sophisticated forms of feedback for learning (e.g., formative feedback). Beyond demonstrating efficacy, future research may also examine the effectiveness of LLMs in providing feedback. The publicly available LLMs (i.e., ChatGPT and Claude) were sufficient to provide criterion-based marking in our analysis, which could output standard educational resources as indicated by our preliminary comparison with a teacher representative. Given that the lack of high-quality feedback has long-term influences^18^, the integration of LLMs offers a promising solution to this enduring educational challenge.

This study also has some limitations. Firstly, the domain-specific information provided to ChatGPT was limited to those available on the IELTS official website. This approach might neglect essential knowledge used by IELTS examiners, leading to a low interrater agreement. Second, the limited number of sample essays could affect the precision of our ICC estimates. +

Overall, this study offers empirical support for using LLM-generated educational feedback. The observed good interrater and intrarater consistency on the IELTS writing task suggests that LLMs (e.g., ChatGPT and Claude) can provide criterion-based grading. However, the gap between interrater agreement and consistency underscores the need for careful implementation and the importance of detailed criteria for enhancing feedback quality in broad assessments.

## Methods

### Essay

This study used sample answers derived from the IELTS book series, spanning from IELTS 8 to IELTS 18. The chosen essays met specific criteria: (1) originating from the academic writing task 2 in IELTS and (2) having been assigned a score by an official IELTS examiner. In total, 31 essays were included in the study, with a mean score of 6.0 and a standard deviation of 1.1, ranging from 3.5 to 8.0. These essays and their corresponding writing prompts were extracted for subsequent analysis.

### ChatGPT prompt

To systematically assess the impact of criteria knowledge on ChatGPT’s grading, we employed a three-stage incremental prompt design. Prompt 1 adhered to best practices for interacting with ChatGPT by instructing it to simulate an IELTS examiner using zero-shot reasoning. Building upon this initial setup, Prompt 2 introduced the official IELTS grading criteria – namely “task response,” “coherence and cohesion,” “lexical resource,” and “grammatical range and accuracy”. Prompt 3 expanded on Prompt 2 by incorporating comprehensive band descriptors for each criterion. This progressive approach allowed us to assess how varying levels of criterion knowledge influence the alignment of LLMs with criterion-based grading. Detailed descriptions of each prompt are available in the supplementary note 1-3.

### Procedure

Initial assessments were conducted using ChatGPT 3.5. Each essay was evaluated in a new chat session to prevent potential influence from chat history. Since Prompt 3 exceeded the maximum word count for a single chat input, the ChatGPT PROMPTs Splitter (https://chatgpt-prompt-splitter.jjdiaz.dev/) was used to segment the prompt. If ChatGPT’s responses did not conform to the IELTS scoring guidelines (e.g., rounding scores to the nearest whole or half band score), the respective essay was re-evaluated until a compliant score was provided.

After identifying the most effective prompt condition, we reran it using ChatGPT 4 to assess whether more advanced models (e.g., more model parameters) can improve the grading. To testify the generalizability of the results to other LLMs, the most effective prompt was also conducted in Claude 3 Haiku, another publicly available LLM with fewer model parameters but a more recent knowledge base.

As the preceding analyses primarily focused on the efficacy of LLM-generated grading, a preliminary assessment of its effectiveness was conducted by having a high school English teacher from China evaluate the same set of essays. The invited teacher, who held general qualifications in English education but lacked specific IELTS training or teaching experience, provided grading that reflects the typical feedback an English learner might receive from a general educational resource. This grading provides an initial reference for contextualizing the effectiveness of our results.

### Statistical analysis

Three pairwise ICC analyses were first conducted, each involving one ChatGPT rating given by one of the prompts and the official rating. The two-way random model (also known as ICC2) and two-way mixed model (also known as ICC3) were conducted. Compared to ICC3, ICC2 assumes fixed biases, which gives rise to the distinction – ICC2 gauges the absolute agreement, while ICC3 primarily assesses consistency^17^. Evaluating ICC2 and ICC3 together offers insights into potential biases^17^. ICCs were conducted using the single-score formula. The point estimates and their 95% confidence intervals were reported.

We initially examined whether each prompt demonstrated significant absolute agreement, defined by a 95% confidence interval that excludes 0. The prompt that generated significant absolute agreement was subjected to follow-up analyses. The values of ICC2 and ICC3 were first inspected. In the presence of a discrepancy between ICC2 and ICC3, indicating potential biases, the Bland-Altman plot and t-tests were conducted to examine the distribution and the tendency of the biases^19^.

After identifying the most effective prompt, we assessed its test-retest reliability (i.e., the intrarater agreement) by rerunning the prompt using ChatGPT 3.5 and applying the average-score ICC2 formula. Subsequently, we extended our analysis by rerunning this prompt with ChatGPT 4.0 and Claude 3.0 Haiku, as well as having a representative secondary middle school English teacher from China evaluate the same set of essays. The ICCs were calculated for each condition. To determine whether these ICCs differed significantly from those of the initial ChatGPT 3.5 grading, we applied Fisher’s Z transformation and conducted statistical comparisons.

ICC qualitative interpretation was guided by Koo & Li^20^: scores below 0.5 are considered poor, 0.5-0.75 moderate, 0.75-0.9 good, and above 0.90 excellent.

## Supplementary information

Supplementary Information

## Data Availability

The data that support the findings of this study are available from the corresponding author upon reasonable request.
