# Supplementary material for: Evaluating large language models for criterion-based grading from agreement to consistency
Source: NPJ Sci Learn. 2024 Dec 30;9:79. doi: 10.1038/s41539-024-00291-1 (PMC11683144; doi:10.1038/s41539-024-00291-1)
Supplement: Supplementary file 1 — Supplementary Information [file 41539_2024_291_MOESM1_ESM.pdf]

Supplementary Note 1

Prompt 1

Act as an IELTS examiner. Before responding, read through the entire prompt. On a scale of 1 to 9, evaluate and grade the following essay in relation to a given prompt.

Essay Prompt:

Essay:

Let's think step by step.

Supplementary Note 2

Prompt 2

Act as an IELTS examiner. Before responding, read through the entire prompt. On a scale of 1 to 9, evaluate and grade the following essay in relation to a given prompt.

The grade should be based on the following four criteria.

1. Task response. This criterion requires the test takers to formulate and develop a position in relation to a given prompt in the form of a question or statement. Ideas should be supported by evidence and examples may be drawn from from the test takers' own experience. Test takers are penalized if they don't cover all of the points in the question. Responses must be at least 250 words in length.
2. Coherence and cohesion. This criterion is concerned with the overall clarity and fluency of the message: how the response organises and links information, ideas and language. Coherence refers to the linking of ideas through logical sequencing. Cohesion refers to the varied and appropriate use of cohesive devices (for example, logical connectors, pronouns and conjunctions) to assist in making the conceptual and referential relationships between and within sentences clear.
3. Lexical resource. This criterion refers to the range of vocabulary the test taker has used and the accuracy and appropriacy of that use in terms of the specific task.
4. Grammatical range and accuracy. This criterion refers to the range and accurate use of the test taker's grammatical resource as manifested in the test taker's writing at the sentence level.

Award a band score for each of the four criteria on a scale of 1 to 9. The four criteria are weighted equally. The overall band score is the average of the four section scores, rounded to the nearest whole or half band. If the average of the four sections ends in .25, the overall band score is rounded up to the next half band, and if it ends in .75, the overall band score is rounded up to the next whole band.

Essay Prompt:

Essay:

Let's think step by step.

Supplementary Note 3

Prompt 3

Act as an IELTS examiner. Before responding, read through the entire prompt. On a scale of 1 to 9, evaluate and grade the following essay in relation to a given prompt.

The grade should be based on the following four criteria.

1. Task response. This criterion requires the test takers to formulate and develop a position in relation to a given prompt in the form of a question or statement. Ideas should be supported by evidence and examples may be drawn from from the test takers' own experience. Test takers are penalized if they don't cover all of the points in the question. Responses must be at least 250 words in length.

   Here are the band descriptors for the task response:

Band 9 - The prompt is appropriately addressed and explored in depth. A clear and fully developed position is presented which directly answers the question/s. Ideas are relevant, fully extended and well supported. Any lapses in content or support are extremely rare.

Band 8 - The prompt is appropriately and sufficiently addressed. A clear and well-developed position is presented in response to the question/s. Ideas are relevant, well extended and supported. There may be occasional omissions or lapses in content.

Band 7 - The main parts of the prompt are appropriately addressed. A clear and developed position is presented. Main ideas are extended and supported but there may be a tendency to over-generalise or there may be a lack of focus and precision in supporting ideas/material.

Band 6 - The main parts of the prompt are addressed (though some may be more fully covered than others). An appropriate format is used. A position is presented that is directly relevant to the prompt, although the conclusions drawn may be unclear, unjustified or repetitive. Main ideas are relevant, but some may be insufficiently developed or may lack clarity, while some supporting arguments and evidence may be less relevant or inadequate.

Band 5 - The main parts of the prompt are incompletely addressed. The format may be inappropriate in places. The writer expresses a position, but the development is not always clear . Some main ideas are put forward, but they are limited and are not sufficiently developed and/or there may be irrelevant detail. There may be some repetition.

Band 4 - The prompt is tackled in a minimal way, or the answer is tangential, possibly due to some misunderstanding of the prompt. The format may be inappropriate. A position is discernible, but the reader has to read carefully to find it. Main ideas are difficult to identify and such ideas that are identifiable may lack relevance, clarity and/or support. Large parts of the response may be repetitive.

Band 3 - No part of the prompt is adequately addressed, or the prompt has been misunderstood. No relevant position can be identified, and/or there is little direct response to the question/s. There are few ideas, and these may be irrelevant or insufficiently developed.

Band 2 - The content is barely related to the prompt. No position can be identified. There may be glimpses of one or two ideas without development.

Band 1 - Responses of 20 words or fewer are rated at Band 1. The content is wholly unrelated to the prompt. Any copied rubric must be discounted.

2. Coherence and cohesion. This criterion is concerned with the overall clarity and fluency of the message: how the response organises and links information, ideas and language. Coherence refers to the linking of ideas through logical sequencing. Cohesion refers to the varied and appropriate use of cohesive devices (for example, logical connectors, pronouns and conjunctions) to assist in making the conceptual and referential relationships between and within sentences clear.

   Here are the band descriptors for the coherence and cohesion:

Band 9 - The message can be followed effortlessly. Cohesion is used in such a way that it very rarely attracts attention. Any lapses in coherence or cohesion are minimal. Paragraphing is skilfully managed.

Band 8 - The message can be followed with ease. Information and ideas are logically sequenced, and cohesion is well managed. Occasional lapses in coherence and cohesion may occur. Paragraphing is used sufficiently and appropriately.

Band 7 - Information and ideas are logically organised, and there is a clear progression throughout the response. (A few lapses may occur, but these are minor.) A range of cohesive devices including reference and substitution is used flexibly but with some inaccuracies or some

over/under use. Paragraphing is generally used effectively to support overall coherence, and the sequencing of ideas within a paragraph is generally logical.

Band 6 - Information and ideas are generally arranged coherently and there is a clear overall progression. Cohesive devices are used to some good effect but cohesion within and/or between sentences may be faulty or mechanical due to misuse, overuse or omission. The use of reference and substitution may lack flexibility or clarity and result in some repetition or error. Paragraphing may not always be logical and/or the central topic may not always be clear.

Band 5 - Organisation is evident but is not wholly logical and there may be a lack of overall progression. Nevertheless, there is a sense of underlying coherence to the response. The relationship of ideas can be followed but the sentences are not fluently linked to each other. There may be limited/overuse of cohesive devices with some inaccuracy. The writing may be repetitive due to inadequate and/or inaccurate use of reference and substitution. Paragraphing may be inadequate or missing.

Band 4 - Information and ideas are evident but not arranged coherently and there is no clear progression within the response. Relationships between ideas can be unclear and/or inadequately marked. There is some use of basic cohesive devices, which may be inaccurate or repetitive. There is inaccurate use or a lack of substitution or referencing. There may be no paragraphing and/or no clear main topic within paragraphs.

Band 3 - There is no apparent logical organisation. Ideas are discernible but difficult to relate to each other. There is minimal use of sequencers or cohesive devices. Those used do not necessarily indicate a logical relationship between ideas. There is difficulty in identifying referencing. Any attempts at paragraphing are unhelpful.

Band 2 - There is little relevant message, or the entire response may be off-topic. There is little evidence of control of organisational features.

Band 1 - Responses of 20 words or fewer are rated at Band 1. The writing fails to communicate any message and appears to be by a virtual non-writer.

3. Lexical resource. This criterion refers to the range of vocabulary the test taker has used and the accuracy and appropriacy of that use in terms of the specific task.

Here are the band descriptors for the lexical resource:

Band 9 - Full flexibility and precise use are widely evident. A wide range of vocabulary is used accurately and appropriately with very natural and sophisticated control of lexical features. Minor errors in spelling and word formation are extremely rare and have minimal impact on communication.

Band 8 - A wide resource is fluently and flexibly used to convey precise meanings. There is skilful use of uncommon and/or idiomatic items when appropriate, despite occasional inaccuracies in word choice and collocation. Occasional errors in spelling and/or word formation may occur, but have minimal impact on communication.

Band 7 - The resource is sufficient to allow some flexibility and precision. There is some ability to use less common and/or idiomatic items. An awareness of style and collocation is evident, though inappropriacies occur. There are only a few errors in spelling and/or word formation and they do not detract from overall clarity.

Band 6 - The resource is generally adequate and appropriate for the task. The meaning is generally clear in spite of a rather restricted range or a lack of precision in word choice. If the writer is a risk-taker, there will be a wider range of vocabulary used but higher degrees of inaccuracy or inappropriacy. There are some errors in spelling and/or word formation, but these do not impede communication.

Band 5 - The resource is limited but minimally adequate for the task. Simple vocabulary may be used accurately but the range does not permit much variation in expression. There may be frequent lapses in the appropriacy of word choice and a lack of flexibility is apparent in frequent simplifications and/or repetitions. Errors in spelling and/or word formation may be noticeable and may cause some difficulty for the reader.

Band 4 - The resource is limited and inadequate for or unrelated to the task. Vocabulary is basic and may be used repetitively. There may be inappropriate use of lexical chunks (e.g. memorised phrases, formulaic language and/or language from the input material). Inappropriate word choice and/or errors in word formation and/or in spelling may impede meaning.

Band 3 - The resource is inadequate (which may be due to the response being significantly underlength). Possible over-dependence on input material or memorised language. Control of

word choice and/or spelling is very limited, and errors predominate. These errors may severely impede meaning.

Band 2 - The resource is extremely limited with few recognisable strings, apart from memorised phrases. There is no apparent control of word formation and/or spelling.

Band 1 - Responses of 20 words or fewer are rated at Band 1. No resource is apparent, except for a few isolated words.

4. Grammatical range and accuracy. This criterion refers to the range and accurate use of the test taker's grammatical resource as manifested in the test taker's writing at the sentence level.

   Here are the band descriptors for the grammatical range and accuracy:

Band 9 - A wide range of structures is used with full flexibility and control. Punctuation and grammar are used appropriately throughout. Minor errors are extremely rare and have minimal impact on communication.

Band 8 - A wide range of structures is flexibly and accurately used. The majority of sentences are error-free, and punctuation is well managed. Occasional, non-systematic errors and inappropriacies occur, but have minimal impact on communication.

Band 7 - A variety of complex structures is used with some flexibility and accuracy. Grammar and punctuation are generally well controlled, and error-free sentences are frequent. A few errors in grammar may persist, but these do not impede communication.

Band 6 - A mix of simple and complex sentence forms is used but flexibility is limited. Examples of more complex structures are not marked by the same level of accuracy as in simple structures. Errors in grammar and punctuation occur, but rarely impede communication.

Band 5 - The range of structures is limited and rather repetitive. Although complex sentences are attempted, they tend to be faulty, and the greatest accuracy is achieved on simple sentences. Grammatical errors may be frequent and cause some difficulty for the reader. Punctuation may be faulty.

Band 4 - A very limited range of structures is used. Subordinate clauses are rare and simple sentences predominate. Some structures are produced accurately but grammatical errors are frequent and may impede meaning. Punctuation is often faulty or inadequate.

Band 3 - Sentence forms are attempted, but errors in grammar and punctuation predominate (except in memorised phrases or those taken from the input material). This prevents most meaning from coming through. Length may be insufficient to provide evidence of control of sentence forms.

Band 2 - There is little or no evidence of sentence forms (except in memorised phrases).

Band 1 - Responses of 20 words or fewer are rated at Band 1. No rateable language is evident.

Award a band score for each of the four criteria on a scale of 1 to 9. The four criteria are weighted equally. The overall band score is the average of the four section scores, rounded to the nearest whole or half band. If the average of the four sections ends in .25, the overall band score is rounded up to the next half band, and if it ends in .75, the overall band score is rounded up to the next whole band.

Essay Prompt:

Essay:

Let's think step by step.
